# In Silico and In Vivo Studies on the Mechanisms of Chinese Medicine Formula (Gegen Qinlian Decoction) in the Treatment of Ulcerative Colitis

**DOI:** 10.3389/fphar.2021.665102

**Published:** 2021-06-11

**Authors:** Xiaolu Liu, Yuling Fan, Lipeng Du, Zhigang Mei, Yang Fu

**Affiliations:** ^1^ Institute of Basic Theory for Integrated Traditional Chinese and Western Medicine, College of Integrated Traditional Chinese and Western Medicine, Hunan University of Chinese Medicine, Changsha, China; ^2^ Third-Grade Pharmacological Laboratory on Chinese Medicine Approved by State Administration of Traditional Chinese Medicine, Medical College of China Three Gorges University, Yichang, China; ^3^ Xiangyang Hospital of Traditional Chinese Medicine, Xiangyang, China

**Keywords:** gegen qinlian decoction (GQD), network pharmacology, molecular docking, inflammatory bowel disease, ulcerative colitis

## Abstract

Ulcerative colitis (UC) is a chronic inflammatory bowel disease, and Gegen Qinlian Decoction (GQD), a Chinese botanical formula, has exhibited beneficial efficacy against UC. However, the mechanisms underlying the effect of GQD still remain to be elucidated. In this study, network pharmacology approach and molecular docking in silico were applied to uncover the potential multicomponent synergetic effect and molecular mechanisms. The targets of ingredients in GQD were obtained from Traditional Chinese Medicine Systems Pharmacology Database and Analysis Platform (TCMSP) and Bioinformatics Analysis Tool for Molecular mechANism of TCM (BATMAN-TCM) database, while the UC targets were retrieved from Genecards, therapeutic target database (TTD) and Online Mendelian Inheritance in Man (OMIM) database. The topological parameters of Protein-Protein Interaction (PPI) data were used to screen the hub targets in the network. The possible mechanisms were investigated with gene ontology (GO) enrichment analysis and Kyoto Encyclopedia of Genes and Genomes (KEGG) pathway enrichment analysis. Molecular docking was used to verify the binding affinity between the active compounds and hub targets. Network pharmacology analysis successfully identified 77 candidate compounds and 56 potential targets. The targets were further mapped to 20 related pathways to construct a compound-target-pathway network and an integrated network of GQD treating UC. Among these pathways, PI3K-AKT, HIF-1, VEGF, Ras, and TNF signaling pathways may exert important effects in the treatment of UC via inflammation suppression and anti-carcinogenesis. In the animal experiment, treatment with GQD and sulfasalazine (SASP) both ameliorated inflammation in UC. The proinflammatory cytokines (TNF-α, IL-1β, and IL-6) induced by UC were significantly decreased by GQD and SASP. Moreover, the protein expression of EGFR, PI3K, and phosphorylation of AKT were reduced after GQD and SASP treatment, and there was no significance between the GQD group and SASP group. Our study systematically dissected the molecular mechanisms of GQD on the treatment of UC using network pharmacology, as well as uncovered the therapeutic effects of GQD against UC through ameliorating inflammation via downregulating EGFR/PI3K/AKT signaling pathway and the pro-inflammatory cytokines such as TNF-α, IL-1β and IL-6.

## Introduction

Ulcerative colitis (UC), a subcategory of inflammatory bowel disease (IBD), is an idiopathic chronic inflammatory disorder of intestinal mucosa or lamina propria (Torres, et al., 2012). The cardinal symptom of this disease can range from mild to severe with many characteristics, including abdominal pain, diarrhea, rectal urgency, and bloody stool (Head and Jurenka, 2003). In recent years, the overall incidence and prevalence of UC is reported to be increased per year (Shivashankar et al., 2017). However, the occurrence and development of UC are connected to the etiology and pathology of multiple factors in human body, such as age, sex, genetics, environmental factors and autoimmunity, which makes UC a complex disease (Ordás et al., 2012). Notably, with continuous infiltration of immune cells and repeated stimulation of epithelial cells in intestinal mucosa, a risk of tumor initiation on UC also has dramatically increased (Rubin et al., 2012). Effective treatment is still lacking in clinic, the traditional drug, such as sulfasalazine (SASP) and glucocorticoids, have severe side effects, which restrict their widespread use in clinic. Currently, the options of medication depend on the severity of disease, while 5-Aminosalicylate and corticosteroids are recommended for mild to moderate UC (Bressler et al., 2015). For the patients with moderate to severe UC, calcineurin inhibitors or biologic agent should be selected (Lv et al., 2014). Although there are optimized medical management to choose, some patients still require colectomy, which is an option for decreasing risk of neoplasia and improvement in health-related quality of life (Gallo et al., 2018). Therefore, up to now no regular method is fit for every patient, and patients may require multiple and integral treatment modalities to achieve best therapeutic effect. Fortunately, this happened to coincide with the theory of traditional Chinese medicine (TCM) based on the comprehensive and individual medicinal system.

TCM has been used clinically in Asia for more than 2000 years, and numerous Chinese botanical medicines can be adopted as an auxiliary treatment for UC (Sałaga et al., 2014). Gegen Qinlian decoction (GQD), a well-known Chinese medicinal formula, was originally invented by Zhongjing Zhang, an eminent herbalist in Eastern Han dynasty in China, and it has been clinically applied to treat diarrhea and dysentery for approximately 2000 years. According to the *Chinese pharmacopoeia*, GQD consists of four botanical drugs, namely *Pueraria lobata (Willd.) Ohwi* (Ge-Gen in Chinese, GG), *Scutellaria baicalensis Georgi* (Huang-Qin in Chinese, HQ), *Coptis chinensis Franch* (Huang-Lian in Chinese, HL), and *Glycyrrhiza uralensis Fisch* (Gan-Cao in Chinese, GC) at the ration of 8:3:3:2. Our previous study and clinical studies have revealed that GQD possessed amazing curative effects in the treatment of UC (Shijun, 2010; Yan et al., 2012; Fan et al., 2019). However, researches on GQD were limited to single pharmacological activity, such as alleviating the gastrointestinal function, anti-inflammatory and antibacterial properties, the overall relationships between compounds and pharmacological mechanisms of GQD have not been clarified in depth (Yu et al., 2005; Xu et al., 2015). Systems network pharmacology is a newly prominent field that combines multiple disciplines and techniques and attempts to probe potential molecular mechanisms and relationships by constructing biological network models (Huang et al., 2014; Kim et al., 2018a). At present, the network pharmacology analysis has been largely applied for several TCM formulae pharmacological research such as the Sini powder for the treatment of chronic hepatitis, the Banxia Xiexin decoction against irritable bowel syndrome, and the antidiabetic activity of GQD in the treatment of type 2 diabetes, as well as the potential mechanisms underlying the formulae effect have also been systematically illuminated (Li et al., 2014; Shu et al., 2018; Li et al., 2019). Thus, in current study, the newly network pharmacology-based approach was employed to integrate active compounds, targets and pathways prediction, and network analysis, which may provide novel insights into the therapeutic effects and molecular mechanisms of GQD. In addition, *in vivo* experiment was also conducted to reveal the underlying mechanisms of GQD in the treatment for UC.

## Materials and Methods

### Chemical Ingredients Database Construction

**FIGURE 1:**
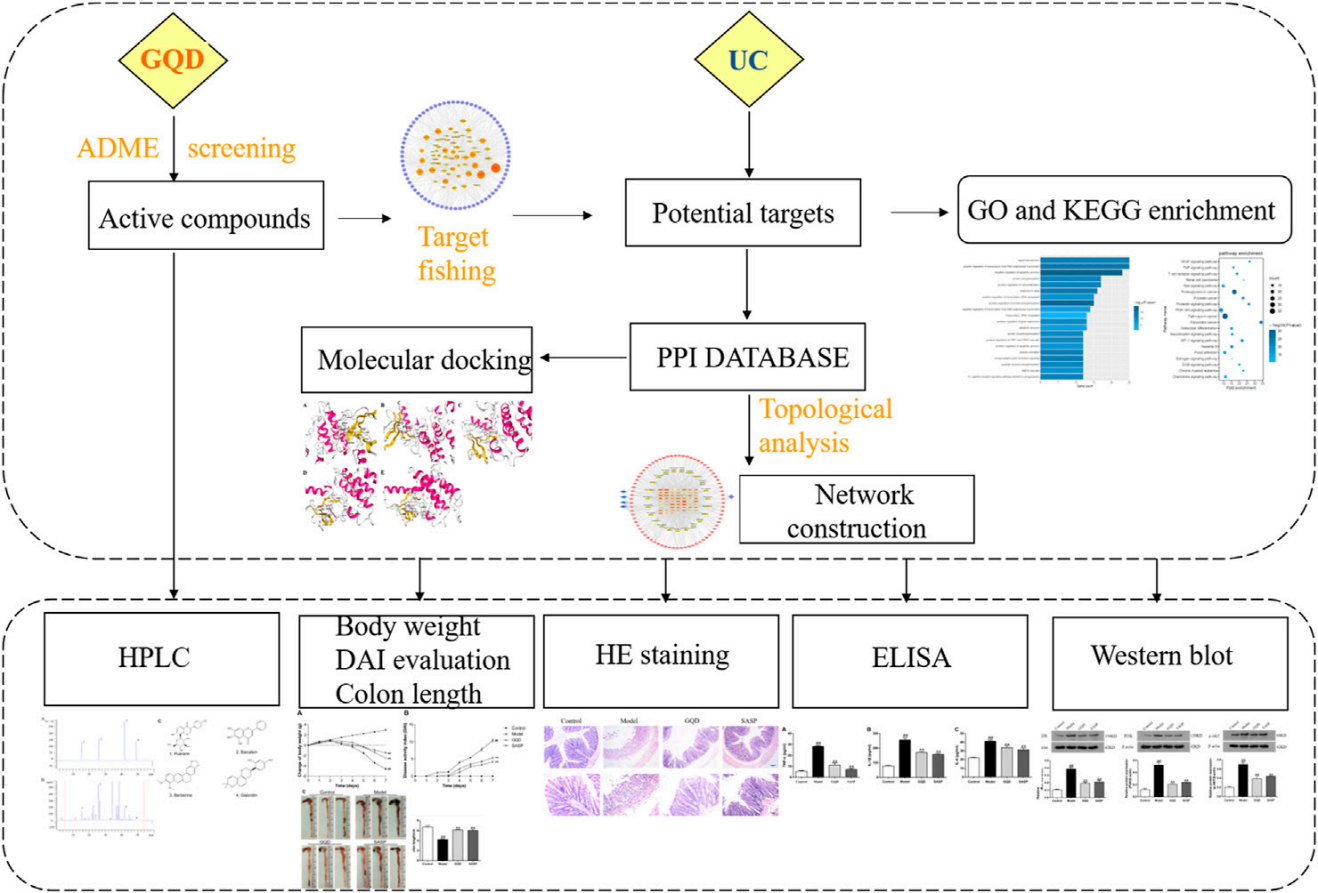
The flowchart of network pharmacology and molecular docking-based strategy for deciphering the underlying mechanisms of GQD on the treatment of UC.

All components of the four Chinese botanical drugs in GQD were retrieved from the Traditional Chinese Medicine Systems Pharmacology Database and Analysis Platform (TCMSP, http://lsp.nwu.edu.cn/) (Ru et al., 2014) and Bioinformatics Analysis Tool for Molecular mechANism of TCM (BATMAN-TCM, http://bionet.ncpsb.org/batman-tcm) (Liu et al., 2016). Then we screen out the compounds which might not be matched with the criterion but were important components by a wide-scale text mining. The framework of this study was shown in Figure 1.

### Pharmacokinetic Selection

To verify the pharmacokinetic characteristics of drugs, a compound screening model provided by the TCMSP data platform, including the evaluation of oral bioavailability (OB), Caco-2 cell permeability (Caco-2) and drug-likeness (DL) were employed. And the three parameters above were clarified as following. OB refers to the rate of an orally administered drugs of unmodified drug that delivers to circulatory system, which is considered predictive of bioactive molecule indicators as therapeutic agents (Xu et al., 2012; Chow, 2014). According to the recommended drug screening criteria, the compounds of which the threshold of OB ≥ 30% in this research could be qualified as a candidate ingredient. Caco-2 is another important parameter generally used as a model to predict the intestinal drug absorption in pre-clinical investigations (Artursson and Karlsson, 1991). And the application of this model employed in screening potential botanical-drug interactions is gaining popularity (Awortwe et al., 2014). In this study, to make sure the putative ingredients of GQD have high permeability via the intestinal membrane enterocytes, we increased the threshold values to Caco-2 ≥ 0.4. DL is a qualitative concept used in drug design for an estimate on how “drug-like” a prospective compound is, which helps to optimize pharmacokinetic and pharmaceutical properties, such as solubility and chemical stability (Tao et al., 2013). The “drug-like” level of the compounds is 0.18, which is used as a selection criterion to filter out molecules with undesirable properties in traditional Chinese botanical drugs (Tian et al., 2015). Consequently, ingredients in database of which the parameters’ information meets the above screening criteria were opted to the next step.

### Potential Targets Fishing

The protein targets of the candidate substances in GQD were retrieved from The Swiss Target Prediction database (http://www.swisstargetprediction.ch/), which can be mined automatically to retrieve specific information for a large number of molecules (Gfeller et al., 2014). In Swiss Target Prediction database, query molecules can be import either as SMILES structure format or drawn in 2D. And the molecular structure information of candidate compounds was derived from PubChem (https://pubchem.ncbi.nlm.nih.gov/), which is a pivotal chemistry resource served as an archive of chemical substance description and structure standardization (Kim et al., 2018b). Then, the target name was mapped to the disease target predictive database to find the disease associated with the protein and retrieved the diseases about UC. UC-related human genes were collected from three resources, which consist of Genecards (http://www.genecards.org), therapeutic target database (TTD) (http://db.idrblab.net/ttd/) and Online Mendelian Inheritance in Man (OMIM) database (http://omim.org/).

### Protein-Protein Interaction

The protein-protein interaction (PPI) of each target were generated from STRING database (http://string-db.org/, ver. 11) with the species limited to “Homo sapiens” and a confidence score > 0.7 (Szklarczyk et al., 2019). Because of the complexity of the original network generated in STRING database, we imported the PPI data into the network visualization software Cytoscape (version 3.7.2) to reconstruct the network for better visualization, and screen out the hub targets.

### Gene Ontology and Kyoto Encyclopedia of Genes and Genomes Pathway Enrichment Analysis

To systematically explore the concerned biological processes of GQD as a therapy against UC, we performed GO and KEGG enrichment analysis by the functional annotation tool DAVID (database for annotation, visualization, and integrated discovery; http://david.abcc.ncifcrf.gov). GO terms and KEGG pathways with *p* value less than 0.05 according to Fisher’s exact test were considered statistically significant and subjected to further analyses.

### Network Construction

To comprehensive understand the molecular mechanisms of GQD to UC, the networks including compound-target-pathway network were constructed using Cytoscape version 3.7.2. The construction of the network was based on the binding of active ingredients to the correlative targets and the signaling pathways, which uncover the underlying interactions between botanical drugs and the disease. In this graphical network, nodes represent the compounds, targets, or signaling pathways by different shapes, and the interactions between them were signified by edges. The degree of a node was marked by different color or size, which was defined as the number of edges relate to it. Moreover, the topological properties were analyzed by the plugin Network Analyzer of Cytoscape (Shannon et al., 2003).

### Molecular Docking

Molecular docking of the critical active compounds with hub targets was performed by CB-Dock (http://cao.labshare.cn/cb-dock/), a web used for predicting the binding sites and affinity with a popular docking program, Autodock Viana (Liu et al., 2020). CB-Dock is a new blind docking tool, which focus on enhancing the docking accuracy. PDB formats of the proteins and a ligand file in the MOL2 or SDF were input to CB-dock to generate a set of points to represent the solvent-accessible surface, and the cavities were ranked according to their sizes. Then a docking box for the following computation was customized, and irrelevant poses was excluded as many as possible. Then we predicted the binding sites, calculated the centers and sizes with a novel curvature-based cavity detection approach, and performed ligand docking with Autodock Vina, and obtained the binding activities and the Vina score, which represents the binding affinity. The more negative the Vina score is, the more stable the ligand-receptor binding (Liu et al., 2020). Ball and stick and cartoon chain represented the ligand and protein, respectively.

### Animals

A total of 60 male C57BL/6 mice weighing about 18–22 g were purchased from the Laboratory Animal Center of China Tree Gorges University (Yichang, China). All mice were randomly assigned to four groups (*n* = 15/group): control group, model group, GQD group and SASP group. All animals were maintained under a 12 h light/dark cycle environment. The room temperature was controlled at (22 ± 1)°C, with (60 ± 5) % humidity, and adequate food and water were provided. The animal experiments were approved by the Laboratory Animal Ethical Committee of Three Gorges University, China (No. 20180904C, approved at 2018-9-11).

### Drugs and Reagents

The botanical formula GQD is comprised of four medicinal botanical drugs: *Pueraria lobata (Willd.) Ohwi* (Batch No: 202011027, Hubei Tianji Pharmaceutical Co. Ltd.), *Scutellaria baicalensis Georgi* (Batch No: 20201103, Hubei Tianji Pharmaceutical Co. Ltd.), *Coptis chinensis Franch* (Batch No: 202011019, Hubei Tianji Pharmaceutical Co. Ltd.), and *Glycyrrhiza uralensis Fisch* (Batch No: 202012018, Hubei Tianji Pharmaceutical Co. Ltd.). All the botanical drugs were purchased from Yichang Hospital of Traditional Chinese Medicine, Hubei province, China. Dextran sulfate sodium (DSS; molecular weight: 36–50 kDa) was obtained from MP Biomedicals (Santa Ana, CA, United States). EGFR antibody, and PI3K antibody were obtained from Proteintech Group (Chicago, United States); *p*-AKT antibody was purchased from Affinity Biologicals (Yarker, Canada), and β-actin antibody was obtained from Boster Biological Technology (Wuhan, China).

### Preparation of Gegen Qinlian Decoction Extract

For the extraction of GQD, *Pueraria lobata (Willd.) Ohwi* (24 g), *Scutellaria baicalensis Georgi* (9 g), *Coptis chinensis Franch* (9 g), and *Glycyrrhiza uralensis Fisch* (6 g) were soaked in 75% ethanol at eightfold volume overnight and then boiled for 1.5 h, the first decoction was thus obtained. Then the residue was boiled a second time with an addition of sixfold volume of 75% ethanol for 1 h to obtain the second decoction. Finally, the first and second decoction were mixed, filtered through gauze, and evaporated to dryness under reduced pressure, and then the extractum was freeze drying to powder.

### Component Analysis of Gegen Qinlian Decoction With High-Performance Liquid Chromatography

The analytical standards were purchased from Push Bio-technology Co., Ltd. (Chengdu, China), and the analysis of GQD was performed on Agilent 1,260 Infinity Ⅱ HPLC system (Agilent Technologies, Santa Clara, CA, United States) equipped with a Diode Array Detector (DAD). A reversed phase Welch Ultimate XB C18 (25 × 4.6 mm, 5 μm) was used and kept at 30°C. The mobile phase consisted of aqueous solution of phosphoric acid and methanol with a gradient elution at a flow rate of 1.0 ml/min. And the detection wavelength was set at 250, 350, 280, and 250 nm.

### Treatment Protocol for Dextran Sulfate Sodium-Induced Colitic Mice

Mice were randomly allocated to four groups (*n* = 15/group): control group, model group, GQD group, and SASP group. All mice except the control group were induced by administration of 3% DSS dissolved in drinking water continuously for 7 days. And the mice in normal group received distilled water without DSS throughout the entire experimental period. According to clinical administration dose, the mice in GQD group and SASP group were administered with GQD enema (0.68 g/kg per day) or SASP enema (0.5 g/kg per day), respectively. Meanwhile, the mice in control group and model group were administrated with saline by enemata. The animals were monitored once a day for body weight, stool consistency, and presence of blood in the feces and the anus. At day 7, mice were anesthetized and sacrificed by cervical dislocation, and the colon samples were collected immediately. Then the colon length was measured and stored for subsequently experiments.

### Evaluation of Disease Activity and Colon Length

To assess the severity of colitis, the disease activity index (DAI) was calculated as the average of body weight loss score, diarrhea score, and fecal blood score. DAI evaluation was calculated under the guidance of previously established scoring system (Yan et al., 2018b; Hassan and Hassan, 2018), and the scoring criteria was based on the following parameters: 1) body weight loss (0, no loss; 1, 1–5% loss; 2, 5–10% loss; 3, 10–20% loss; 4, over 20% loss); 2) diarrhea (0, normal; 2, loose stools; 4, watery diarrhea); and 3) hematochezia (0, no bleeding; 2, slight bleeding; 4, gross bleeding). Body weight gain was expressed as the difference from the initial body weight. Colon length was regarded as an indirect marker of inflammation, thereby, the length of colons from the ileocecal junction to the anal verge was measured.

### Histopathological Analysis

Colon tissues were collected and fixed in 4% paraformaldehyde overnight, then dehydrated at gradient concentrations of ethanol, and embedded in paraffin. Tissues were sectioned at 4 μm thickness with a paraffin and stained with hematoxylin and eosin (HE). The images of the tissues were obtained using Image-Pro Plus 5.0 system.

### Enzyme-Linked Immunosorbent Assay

To test the anti-inflammatory effects of GQD on ulcerative colitis, colon homogenate supernatants of all the experimental groups were collected, and the expression of TNF-α, IL-1β, and IL-6 was measured by ELISA kits (USCN Cloud-Clone Corp, Wuhan, China) according to the manufacturer’s instructions.

### Western Blotting Analysis

The total protein of colon tissue was extracted using RIPA lysis buffer with the protease inhibitor cocktail (Beyotime Biotechnology, China), and protein concentration was measured using the BCA protein assay kit (Beyotime Biotechnology) according to the manufacturer’s instructions. Equivalent amounts of protein (20 μg) were separated by SDS-PAGE and then transferred onto 0.45 μm PVDF membranes (Millipore, Billeria, MA, United States). The membranes were then blocked in 5% nonfat milk in TBST buffer for 1 h at room temperature, followed by incubation with primary antibodies at 4°C overnight. Then membranes were washed with TBST and incubated with secondary antibody for 1 h at room temperature, proteins were detected using ECL reagent (Applygen, China). The antibodies used were as follows: EGFR (1:2,000), PI3K (1:1,000), *p*-AKT (1:1,000), and β-actin (1:500).

### Statistical Analysis

All the experimental data were analyzed using SPSS 20.0 statistical software (IBM Corp., Armonk, NY, United States). GraphPad Prism 6.0 software (GraphPad Software Inc., San Diego, CA, United States) was used for the calculations. All results are expressed as means ± standard error of the mean (SEM). Multigroup comparisons were performed using one-way analysis of variance (ANOVA) and post hoc Statistical least significant difference (LSD) test. *p* value < 0.05 was considered statistically significant.

## Results

### Identification of Candidate Compounds in Gegen Qinlian Decoction

After the absorption, distribution, metabolism, elimination (ADME) parameters screening (OB ≥ 30%, DL ≥ 0.18, Caco-2 ≥ 0.4), 75 potential components of the four botanical medicines were collected from database and relate literatures. Among them, 3 ingredients belong to GG, 9 belong to HL, 20 belong to HQ, and 47 belong to GC. It is worth mentioning that formononetin (OB = 69.67%, Caco-2 = 0.78, DL = 0.21) as an important component existed in both GG and GC. Additionally, beta-sitosterol (OB = 39.91%, Caco-2 = 1.32, DL = 0.75), exhibited good ADME properties, can be collected from GG and HQ. Both coptisine (OB = 30.67%, Caco-2 = 1.21, DL = 0.86) and epiberberine (OB = 43.09%, Caco-2 = 1.17, DL = 0.78) are common ingredients found in HQ and HL. Additionally, two compounds, puerarin and daidzein, did not meet the three criteria were taken into consideration as their obvious bioactivities (Qi et al., 2004). Consequently, after removing redundancy, a total of 77 compounds were identified as candidate compounds for further analysis. All the compounds identified from GQD and their concreted predicted OB, DL, and Caco-2 values are showed in Table 1.

**TABLE 1:** Information for the candidate bioactive compounds of GQD.

ID	Chemical	OB (%)	Caco-2	DL	Herb
C01	Puerarin	24.03	−1.15	0.69	GG
C02	Formononetin	69.67	0.78	0.21	GG GC
C03	Daidzein	19.44	0.59	0.19	GG
C04	Beta-sitosterol	36.91	1.32	0.75	GG HQ
C05	3′-methoxydaidzein	48.57	0.56	0.24	GG
C06	Coptisine	30.67	1.21	0.86	HQ HL
C07	Epiberberine	43.09	1.17	0.78	HQ HL
C08	Berberine	36.86	1.24	0.78	HL
C09	Berberrubine	35.74	1.07	0.73	HL
C10	(R)-canadine	55.37	1.04	0.77	HL
C11	Berlambine	36.68	0.97	0.82	HL
C12	Palmatine	64.60	1.33	0.65	HL
C13	Worenine	45.83	1.22	0.87	HL
C14	Moupinamide	86.71	0.55	0.26	Hl
C15	5,2′,6′-trihydroxy-7,8-dimethosyflavone	45.05	0.48	0.33	HQ
C16	5,2′-dihydroxy-6,7,8-trimethoxyflavone	31.71	0.93	0.35	HQ
C17	5,7,4′-trihydroxy-6-methoxyflavanone	36.63	0.43	0.27	HQ
C18	Acacetin	34.97	0.67	0.24	HQ
C19	Baicalein	33.52	0.63	0.21	HQ
C20	Dihydrobaicalin	40.04	0.56	0.21	HQ
C21	Dihydrooroxylin	66.06	0.67	0.23	HQ
C22	Moslosooflavone	44.09	1.01	0.25	HQ
C23	Norwogonin	39.40	0.60	0.21	HQ
C24	Oroxylin a	41.37	0.76	0.23	HQ
C25	Panicolin	76.26	0.84	0.29	HQ
C26	Rivularin	37.94	0.65	0.37	HQ
C27	Salvigenin	49.07	0.86	0.33	HQ
C28	Skullcapflavone Ⅱ	69.51	0.68	0.44	HQ
C29	Stigmasterol	43.83	1.44	0.76	HQ
C30	Supraene	33.55	2.08	0.42	HQ
C31	Wogonin	30.68	0.79	0.23	HQ
C32	1-Methoxyphaseollidin	69.98	1.01	0.64	GC
C33	3′-hydroxy-4′-O-Methylglabrindin	43.71	1.00	0.57	GC
C34	3′-methoxyglabridin	46.16	0.94	0.57	GC
C35	7-Acetoxy-2-methylisoflavone	38.92	0.74	0.26	GC
C36	7-Methoxy-2-methylisoflavone	42.56	1.16	0.20	GC
C37	Calycosin	47.75	0.52	0.24	GC
C38	DFV	32.76	0.51	0.18	GC
C39	Eurycaprin A	43.28	0.43	0.37	GC
C40	Gancaonin A	51.08	0.80	0.40	GC
C41	Gancaonin B	48.79	0.58	0.45	GC
C42	Gancaonin G	60.44	0.78	0.39	GC
C43	Glabranin	52.90	0.97	0.31	GC
C44	Glabrene	46.27	0.99	0.44	GC
C45	Glabridin	53.25	0.97	0.47	GC
C46	Glabrone	52.51	0.59	0.50	GC
C47	Glepidotin A	44.72	0.79	0.35	GC
C48	Glyasperin B	65.22	0.47	0.44	GC
C49	Glyasperin C	45.56	0.71	0.40	GC
C50	Glyasperin F	75.84	0.43	0.54	GC
C51	Glycyrin	52.61	0.59	0.47	GC
C52	Glycyrol	90.78	0.71	0.67	GC
C53	Glypallichalcone	61.60	0.76	0.19	GC
C54	HMO	38.37	0.79	0.21	GC
C55	Inermine	75.18	0.89	0.54	GC
C56	Inflacoumarin A	39.71	0.73	0.33	GC
C57	Isoglycyrol	44.70	0.91	0.84	GC
C58	Isolicoflavonol	45.17	0.54	0.42	GC
C59	Isotrifoliol	31.94	0.53	0.42	GC
C60	Jaranol	50.83	0.61	0.29	GC
C61	Kanzonols W	50.48	0.63	0.52	GC
C62	Licoagrocarpin	58.81	1.23	0.58	GC
C63	Licoagroisoflavone	57.28	0.71	0.49	GC
C64	Licochalcone A	40.79	0.82	0.29	GC
C65	Licochalcone B	76.76	0.47	0.19	GC
C66	Licochalcone G	49.25	0.64	0.32	GC
C67	Licoisoflavone B	38.93	0.46	0.55	GC
C68	Licoricone	63.58	0.53	0.47	GC
C69	Lupiwighteone	51.64	0.68	0.37	GC
C70	Mairin	55.38	0.73	0.78	GC
C71	Medicarpin	49.22	1.00	0.34	GC
C72	Odoratin	49.95	0.42	0.30	GC
C73	Phaseol	78.77	0.76	0.58	GC
C74	Phaseolinisoflavan	32.01	1.01	0.45	GC
C75	Semilicoisoflavone B	48.78	0.45	0.55	GC
C76	Shinpterocarpin	80.30	1.10	0.73	GC
C77	Vestitol	74.66	0.86	0.21	GC

OB, oral bioavailability; Caco-2, Caco-2 cell permeability; DL, drug-likeness.

### Targets Identification of Gegen Qinlian Decoction on Ulcerative Colitis

Based on The Swiss Target Prediction database, a total of 740 targets related to the 77 compounds were obtained in GQD. In order to explore the relevance and underlying mechanism of these targets specific to GQD in the development of UC, A total of 3785 UC-related genes were retrieved from the GeneCards, OMIM, and TTD databases by employing a keyword-based search. After eliminating the overlaps, 287 targets were shared between 740 compound-related targets and 3785 UC-related targets. PPI network of these 287 targets were established in STRING database and visualized by Cytoscape software. There were 287 nodes and 4,490 edges in this interaction network. According to previous study, a node would be defined as a hub target when the degree was more than twofold the median degree of all nodes in the network (Li et al., 2007). Eventually, 59 major hub targets were considered to be the effective therapeutic targets of UC. Topological analysis indicated that serine/threonine-protein kinase AKT (AKT1, degree = 165), vascular endothelial growth factor A (VEGFA, degree = 151), epidermal growth factor receptor erbB1 (EGFR, degree = 141), MAP kinase ERK1 (MAPK3, degree = 141), TNF-alpha (TNF, degree = 136), signal transducer and activator of transcription3 (STAT3, degree = 128), tyrosine-protein kinase SRC (SRC, degree = 126), cyclooxygenase-2 (PTGS2, degree = 113) were the top hub targets based on degree centrality.

### Gene Ontology and Pathway Enrichment Analysis

**FIGURE 2:**
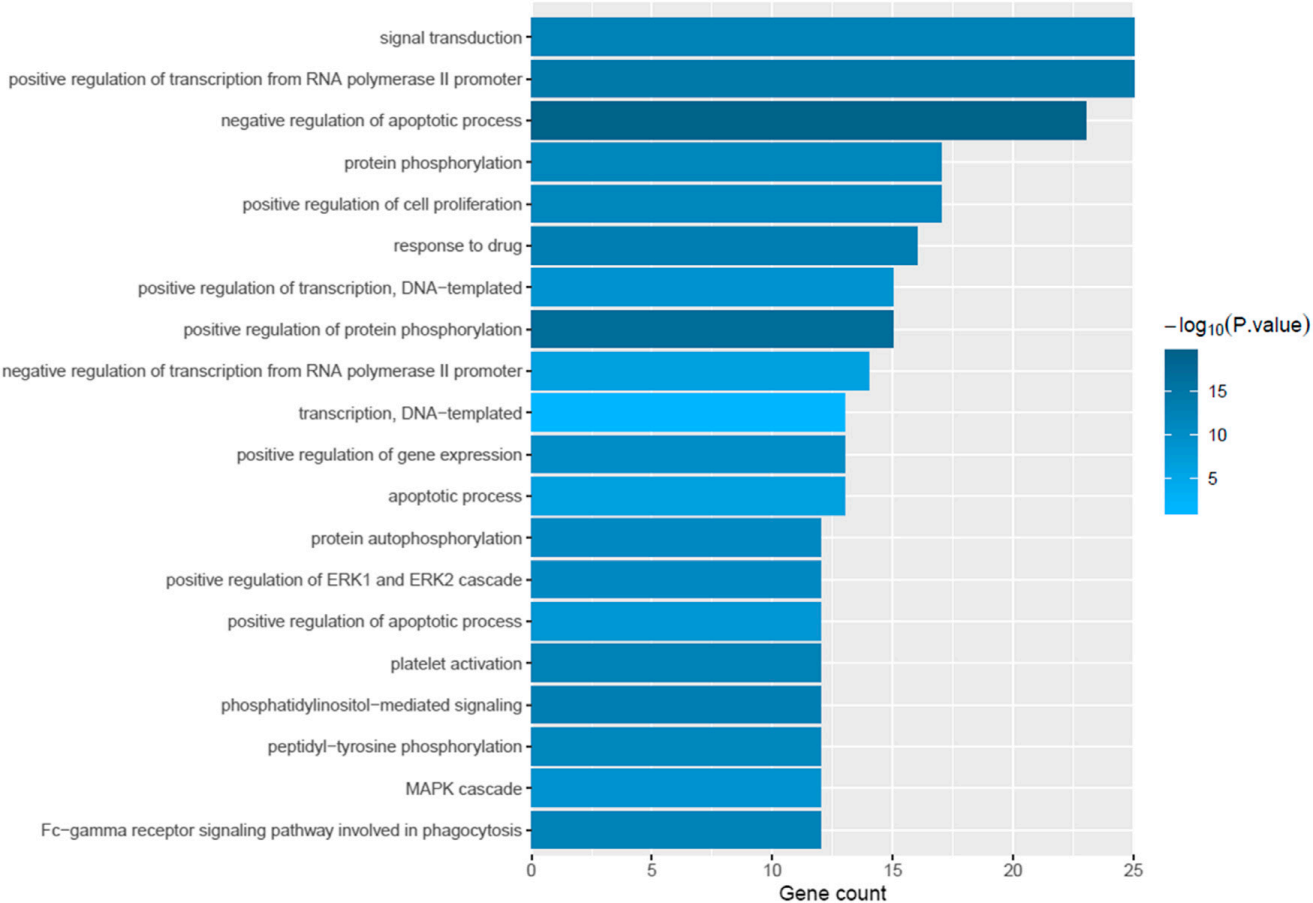
The results of gene ontology (GO) biological process analysis. The X-axis represents gene count, while the Y-axis represents the categories of biological process (*p*-value ≤ 0.05).

**FIGURE 3:**
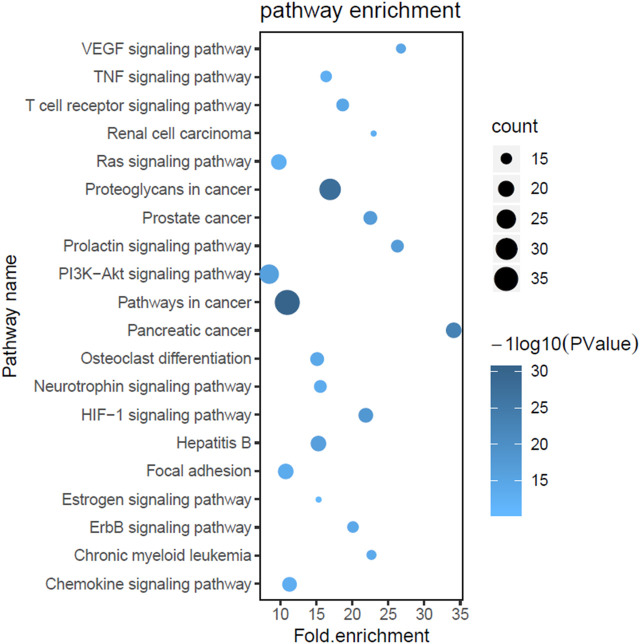
Kyoto Encyclopedia of Genes and Genomes (KEGG) enrichment analysis. The X-axis represents the enrichment rate of these genes in total genes, while the Y-axis represents the enrichment pathways of the target genes (*p*-value ≤ 0.05). The depth of the color represents the size of the value, and the size of circle represents the enrichment counts of these pathways.

To explore the biological processes of the 59 putative targets of GQD on UC, GO and pathway enrichment analysis was further performed. As shown in Figure 2, the top 20 most significantly enriched GO terms were exhibited with their *p-*value and gene count. These targets were obviously enriched in negative regulation of apoptotic process, protein phosphorylation, cell proliferation, MAPK cascade and inflammatory response, which have a strongly correlation with the pathogenesis of UC. These results indicated the multiple synergies of GQD on biological processes. To further reveal the potential mechanisms of the therapeutic effects of GQD, we conducted KEGG pathway enrichment analysis on 59 targets, and screened out 20 pathways based on the threshold of *p*-value < 0.05. The top 20 significantly enriched pathways contained pathways in cancer, HIF-1 signaling pathway, PI3K-AKT signaling pathway, TNF signaling pathway, VEGF signaling pathway and Ras signaling pathway (Figure 3).

### Compound-Target-Pathway Network Analysis

**FIGURE 4:**
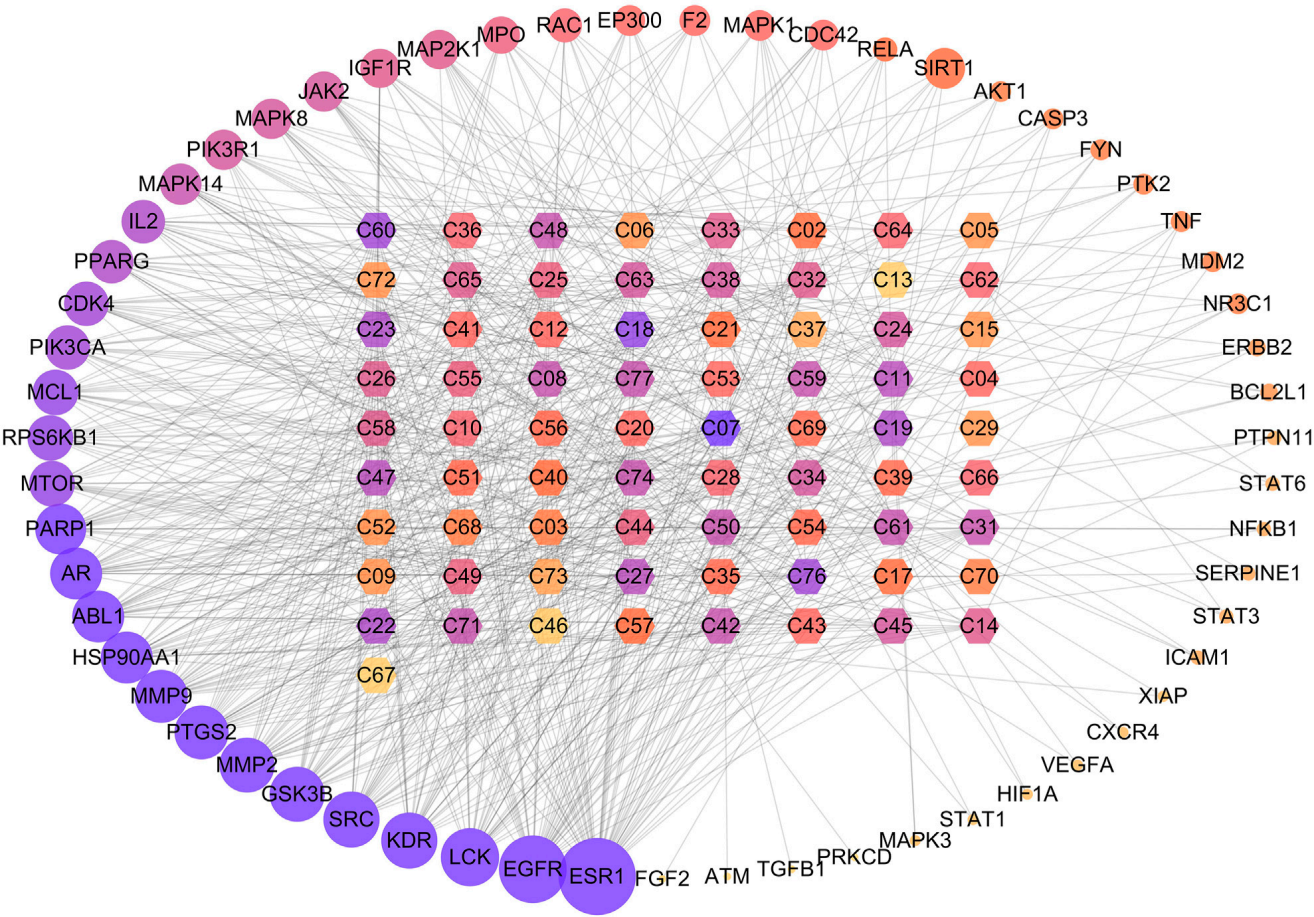
The interaction network between compounds and hub targets. The hexagon represents the herbal compounds, the circles stand for the potential targets, and the edges represent the interactions between them. The depth of color and the size of circle are proportional to their degree value. The ID of the compounds was elaborated in Table 1.

**FIGURE 5:**
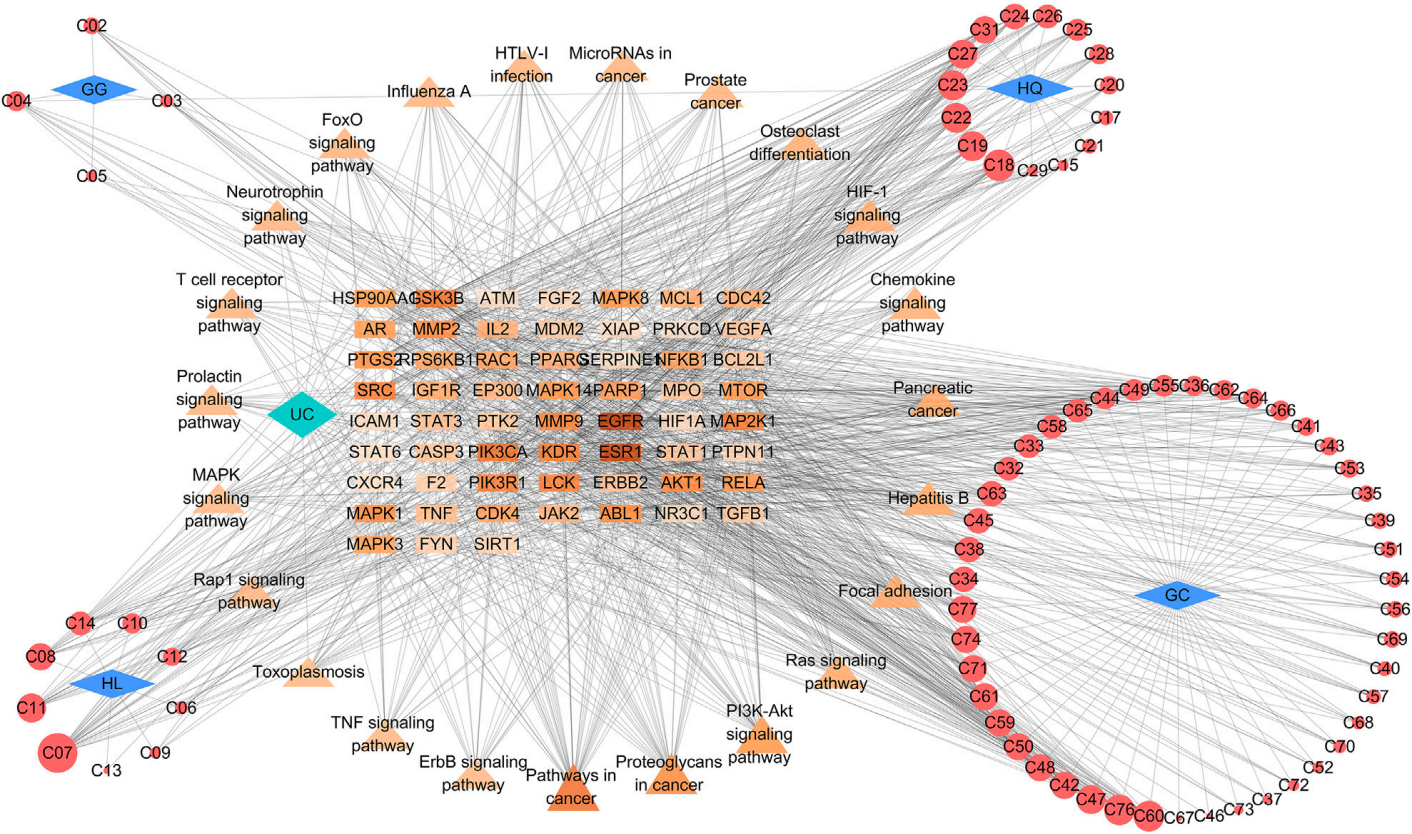
Compound–target–pathway network. The rectangle, triangle and circle represent the potential targets, major pathways and botanical compounds, respectively. The rhombus represents the four botanical drugs in GQD. Edges represent the interaction between them. For the potential targets and pathways, the change in color depth reflect the degree value. For the botanical compounds, the circle size is proportional to their degree value. GG, *Pueraria lobata (Willd.) Ohwi*; HQ, *Scutellaria baicalensis Georgi*; HL, *Coptis chinensis Franch*; GC, *Glycyrrhiza uralensis Fisch*.

To further elucidate the underlying pharmacological mechanisms of the candidate compounds for the treatment of GQD, the compound-target network and compound–target–pathway network were constructed based on the protein targets and the corresponding pathway enrichment analysis. As shown in Figure 4, the compound–target network was composed of 132 nodes and 684 edges, and the average degree value of hub targets was 9.03. The epiberberine (C07, degree = 26) from HQ and HL, acacetin (C18, degree = 18) and the baicalein (C19, degree = 16) from HQ, berberine (C08, degree = 13) from HL, wogonin (C31, degree = 13) from HQ, glabridin (C45, degree = 12) from GC were identified as relatively high-degree active compounds, which indicated that the six compounds play more important roles in the pathological processes of ulcerative colitis. According to previous studies, puerarin, one of the most important constituents in GG, has been widely used in the treatment of UC by inhibiting inflammatory response (Jeon et al., 2020). Therefore, we hypothesize that epiberberine, acacetin, baicalein, berberine, wogonin, glabridin and puerarin may account for the essential therapeutic effects of GQD on UC. Then we selected four representative compounds of each of the four botanical drugs for molecular docking and HPLC analysis. Among these compounds, puerarin from GG, baicalein from HQ, berberine from HL, and glabridin from GC were taken into consideration. In the meantime, the 59 hubs with higher degree scores, such as estrogen receptor alpha (ESR1, degree = 47), epidermal growth factor receptor erbB1 (EGFR, degree = 38), tyrosine-protein kinase SRC (SRC, degree = 27), matrix metalloproteinase 2 (MMP2, degree = 26), glycogen synthase kinase-3 beta (GSK3B, degree = 26), cyclooxygenase-2 (PTGS2, degree = 25) possessed highly connection with compounds and pathways (Figure 5). Detailed information about the hub targets is provided in Table 2. Furthermore, an investigation at the relationship between 59 targets and 20 remarkably enriched pathways was conducted based on the compound-target-pathway network, and the average degree value of hub targets per pathway was 18.39. These pathways probably attributed to the therapeutic effect of GQD on UC.

**TABLE 2:** Potential targets of GQD on UC.

Gene name	Target	Uniprot ID	Degree		
ESR1	Estrogen receptor alpha	P03372	47		
EGFR	Epidermal growth factor receptor erbB1	P00533	38		
LCK	Tyrosine-protein kinase LCK	P06239	29		
SRC	Tyrosine-protein kinase SRC	P12931	27		
KDR	Vascular endothelial growth factor receptor 2	P35968	27		
MMP2	Matrix metalloproteinase 2	P08253	26		
GSK3β	Glycogen synthase kinase-3 beta	P49841	26		
PTGS2	Cyclooxygenase-2	P35354	25		
MMP9	Matrix metalloproteinase 9	P14780	24		
HSP90AA1	Heat shock protein HSP 90-alpha	P07900	23		
AR	Androgen receptor	P10275	23		
ABL1	Tyrosine-protein kinase ABL	P00519	23		
PARP1	Poly [ADP-ribose] polymerase-1	P09874	22		
MTOR	Serine/threonine-protein kinase mTOR	P42345	17		
MCL1	Induced myeloid leukemia cell differentiation protein Mcl-1	Q07820	17		
RPSKB1	Ribosomal protein S6 kinase 1	P23443	17		
PIK3CA	PI-kinase p110-alpha subunit	P42336	16		
CDK4	Cyclin-dependent kinase 4	P11802	16		
PPARG	Peroxisome proliferator-activated receptor gamma	P37231	15		
IL2	Interleukin-2	P60568	14		
MAPK14	MAP kinase p38 alpha	Q16539	13		
MAPK8	c-Jun N-terminal kinase 1	P45983	12		
PIK3R1	PI3-kinase p85-alpha subunit	P27986	12		
JAK2	Tyrosine-protein kinase IAK2	O60674	12		
MAPK2K1	Dual specificity mitogen-activated protein kinase 1	Q02750	11		
IGF1R	Insulin-like growth factor 1 receptor	P08069	11		
MPO	Myeloperoxidase	P05164	10		
RAC1	Ras-related C3 botulinum toxin substrate 1	P63000	9		
MAPK1	MAP kinase ERK2	P28482	8		
EP300	Histone acetyltransferase p300	Q09472	8		
CDC42	Cell division control protein 42 homolog	P60953	8		
F2	Thrombin	P00734	8		
SIRT1	NAD-dependent control protein 42 homolog	Q96EB6	6		
RELA	Nuclear factor NF-kappa-B p65 subunit	Q04206	6
AKT1	Serine/threonine-protein kinase AKT	P31749	5		
TNF	Tumor necrosis factor	P01375	5		
CASP3	Caspase-3	P42574	5		
MDM2	P53-binding protein Mdm-2	Q00987	5		
FYN	Tyrosine-protein kinase fyn	P06241	5		
NR3C1	Glucocorticoid receptor	P04150	5		
PTK2	Focal adhesion kinase 1	Q05397	5		
ERBB2	Receptor protein-tyrosine kinase erbB-2	P04626	4		
BCL2L1	Apoptosis regulator Bcl-X	Q07817	4		
STAT3	Signal transducer and activator pf transcription 3	P40763	3		
ICAM1	Intercellular adhesion molecule-1	P05362	3		
NFκB	Nuclear factor NF-kappa-B p105 subunit	P19838	3		
PTPN11	Protein-tyrosine phosphatase 2C	Q06124	3		
SERPPINE1	Plasminogen activator inhibitor-1	P05121	3		
STAT6	Signal transducer and activator of transcription 6	P42226	3		
VEGFA	Vascular endothelial growth factor A	P15692	2		
MAPK3	MAP kinase ERK1	P27361	2		
CXCR4	C-X-C chemokine receptor type 4	P61073	2		
STAT1	Signal transducer and activator of transcription 1 alpha/beta	P42224	2		
HIF1A	Hypoxia-inducible factor 1 alpha	Q16665	2		
XIAP	Inhibitor of apoptosis protein 3	P98170	2		
FGF2	Basic fibroblast growth factor	P09038	1		
ATM	Serine-protein kinase ATM	Q13315	1		
TGFB1	Transforming growth factor	P01137	1		
PRKCD	Protein kinase C delta	Q05655	1		

### Molecular Docking

**FIGURE 6:**
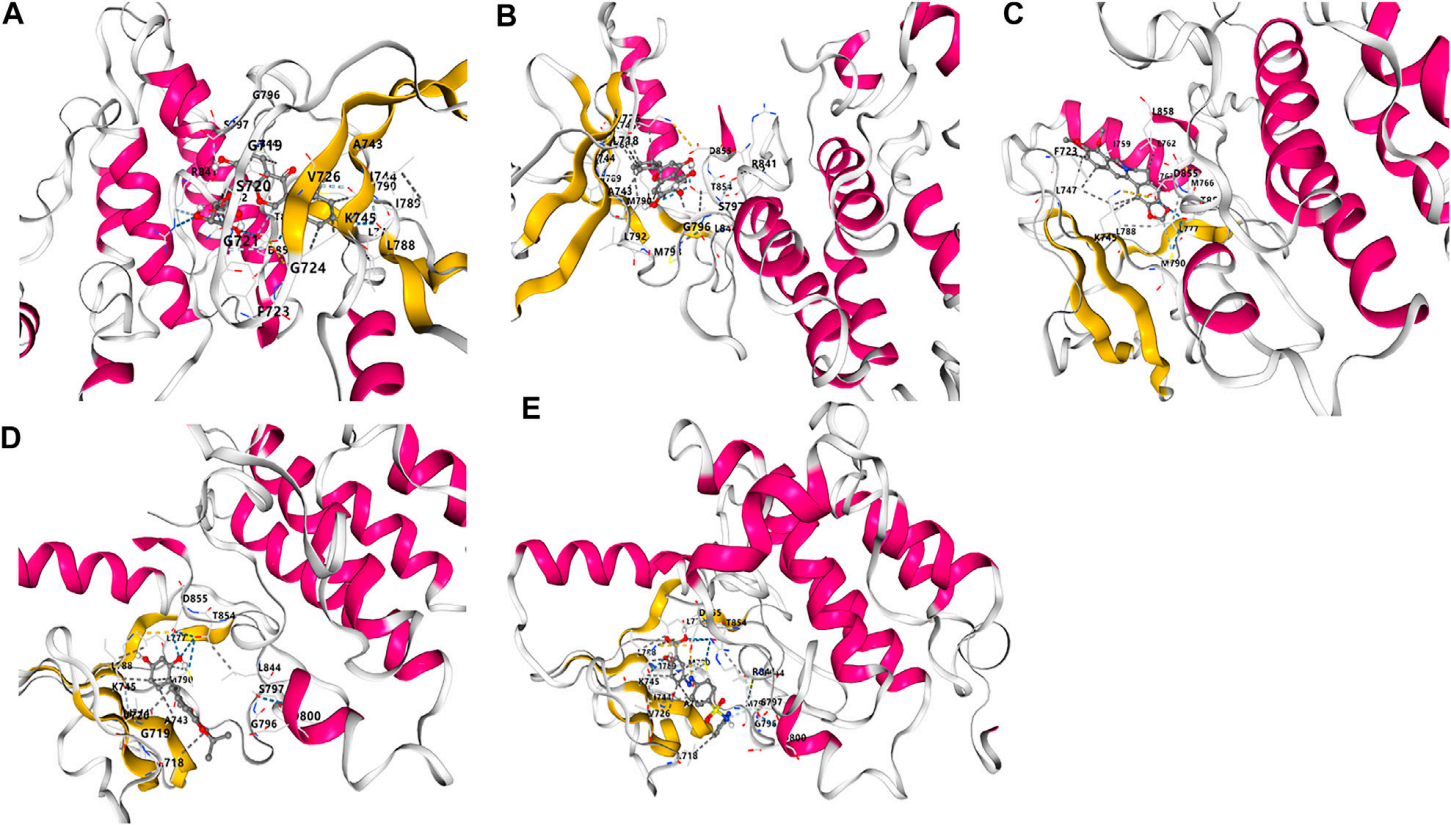
Molecular docking analysis of the binding affinity of the four active compounds toward to the hub target EGFR. **(A)** Puerarin, vina score = −8.4; **(B)** Baicalein, vina score = −7.8; **(C)** Berberine, vina score = −7.8; **(D)** Glabridin, vina score = −7.9. **(E)** SASP, vina score = −8.2.

To further verify the results of network pharmacology screening, the four crucial active compounds in GQD were docked with the hub target EGFR via CB-dock. It is generally believed that the vina score indicates the binding affinity between the ligand and the receptor, and the more negative the vina score is, the more stable the compounds bind to the hub target. As shown in Figure 6, the crucial active components in GQD, which consist of puerarin, baicalein, berberine and glabridin, all possessed good binding activities to EGFR. And puerarin exhibited the best binding affinity with EGFR, which was higher than the positive control drug SASP.

### High-Performance Liquid Chromatography Analysis of Gegen Qinlian Decoction

**FIGURE 7:**
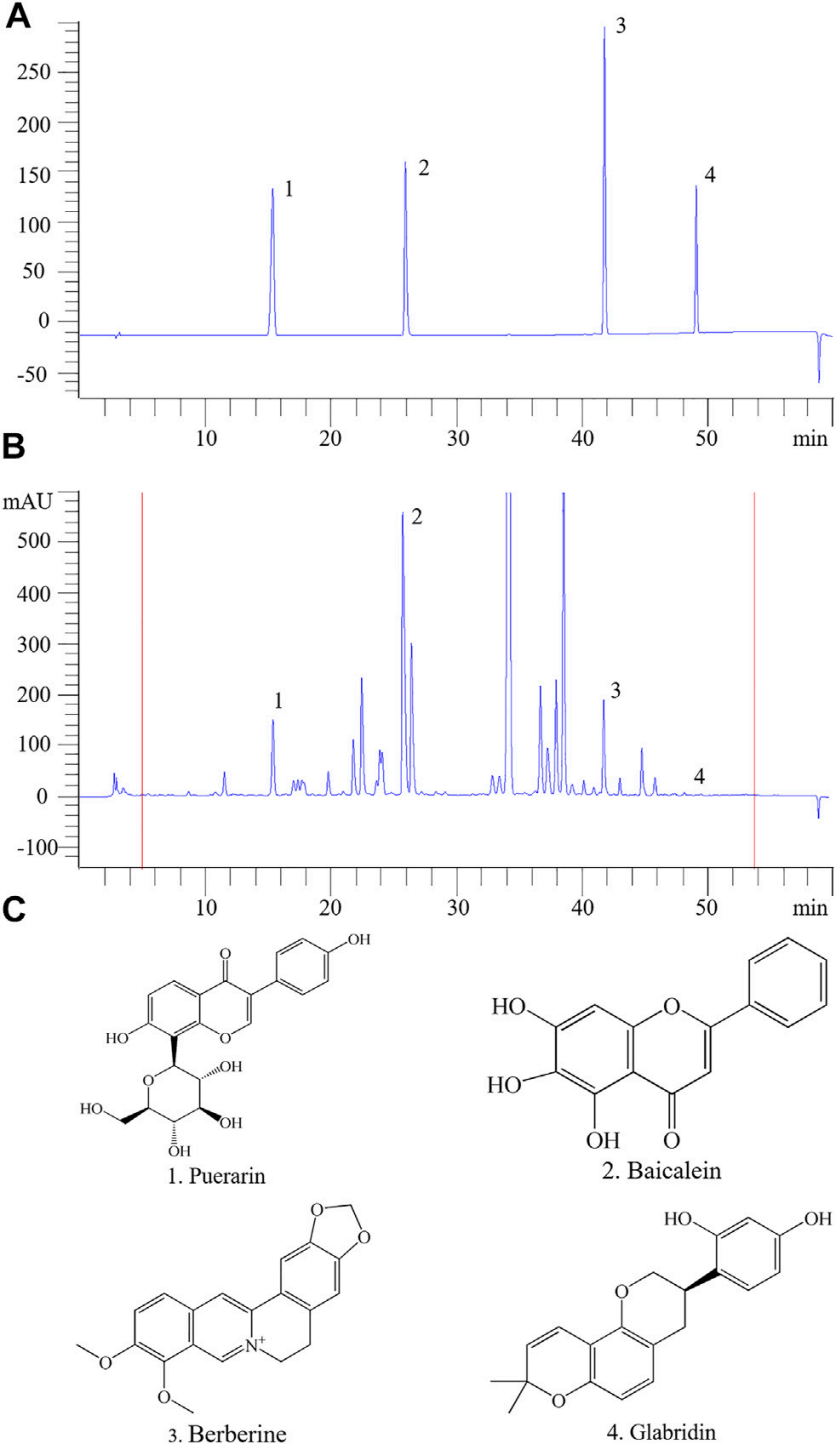
HPLC profiles of the main active components of GQD. **(A)** The mixed standard sample. **(B)** The four active components. **(C)** The chemical formula of the four active compounds. 1. puerarin; 2. baicalein; 3. berberine; 4. glabridin.

As shown in Figure 7, HPLC analysis indicated the retention time of standards puerarin (15.429 min), baicalein (25.764 min), berberine (41.765 min) and glabridin (49.096 min), respectively, in Figure 6A. Moreover, comparison with mixed check samples were performed, and external method was used to calculate the concentrations. The contents of puerarin, baicalein, berberine and glabridin were 1,290.93 μg/g (2.52%), 6,616.57 μg/g (11.35%), 1,129.38 μg/g (2.8%) and 5.49 μg/g (0.57%), respectively. The results showed that GQD contained the four active components. In addition, there were many peaks from more concentrated compounds that were not analyzed quantitatively or qualitatively in the chromatograms of GQD. It has been demonstrated that the glycyrrhizic acid and baicalin, which belong to triterpene saponins and *O*-glycosides, respectively, possess protective effects against non-alcoholic fatty liver disease and diabetes (Wang et al., 2016; Yan et al., 2018a; Qiao et al., 2018). Therefore, the high concentrated compounds in HPLC chromatogram may be the compounds mentioned above.

### Gegen Qinlian Decoction Ameliorated Dextran Sulfate Sodium-Induced Ulcerative Colitis in Mice

**FIGURE 8:**
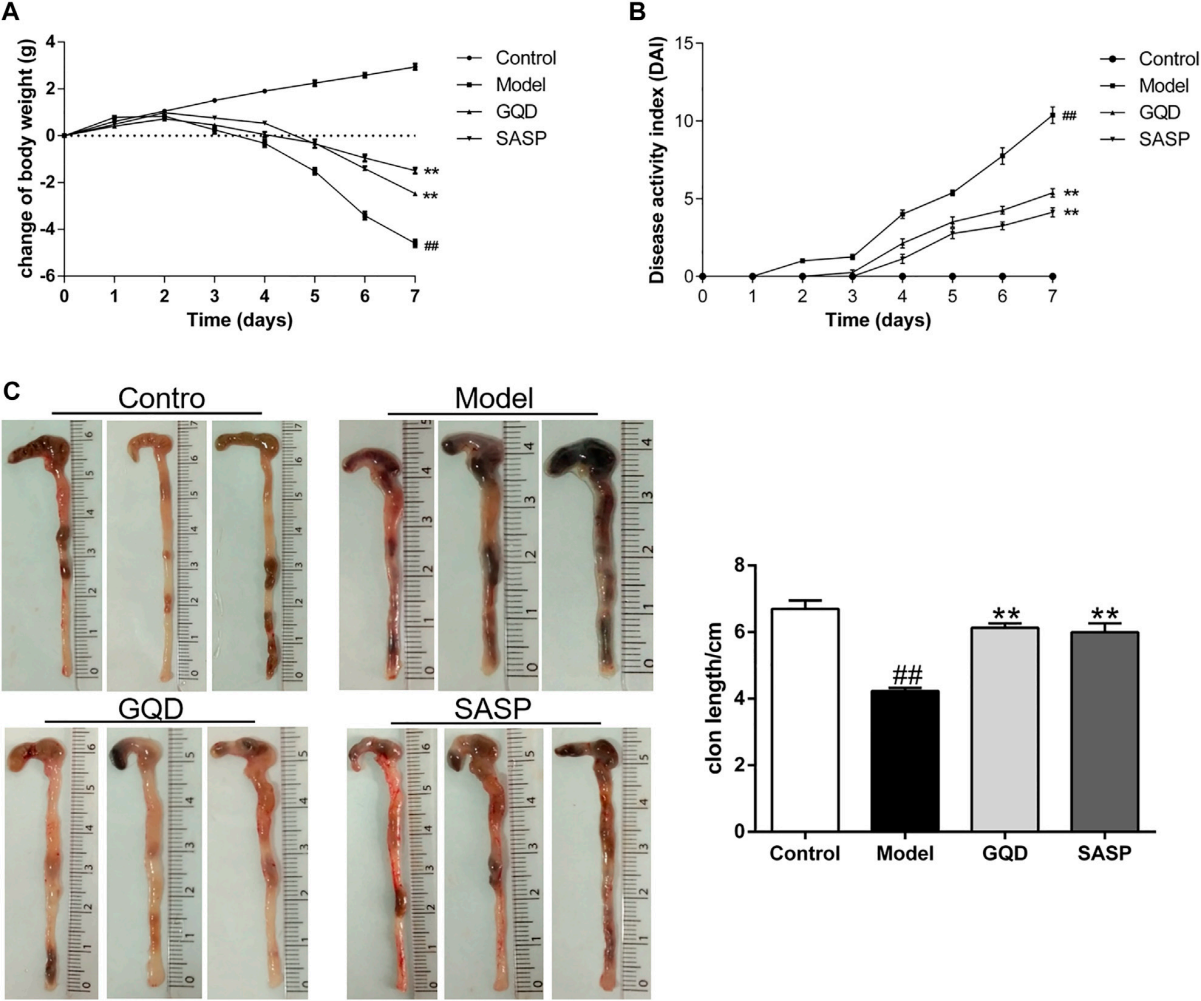
GQD ameliorated DSS-induced body weight loss, DAI score and colonic shortening. **(A)** Change of body weight in four groups. **(B)** DAI score during experimental colitis. **(C)** Colon length in four groups. Values presented as mean ± SEM. ^##^ *p* < 0.01 vs. control; ^**^ *p* < 0.01 vs. model.

To explore the protective effects of GQD on UC, the mouse model of acute ulcerative colitis was established by administrating mice with 5% DSS for 7 days. SASP was served as a positive control in this study. The therapeutic effect of GQD on UC was assessed preliminary by body weight loss, colon length and DAI score. On the 3rd day of DSS induction, body weight of model group was markedly decreased compared to sham group, with a continuous daily decrease thereafter. And administration of GQD and SASP significantly blunted the body weight loss induced by DSS compared with model group (*p* < 0.01, Figure 8A). Meanwhile, DSS administration for 7 days led to severe diarrhea, blood in stool and body weight loss integrated as remarkably high disease activity index compared with normal group (*p* < 0.01), while this elevation was relieved by GQD and SASP treatment (*p* < 0.01, Figure 8B). In addition, after the establishment of UC model, DSS resulted in a significant shortening of the colon length, while GQD and SASP treatment markedly reduced DSS-induced colon shortening compared with model group (*p* < 0.01, Figure 8C). Collectively, we assessed preliminary effect of GQD on UC, and the results suggested that GQD exerted protective effects in UC treatment.

### Effects of Gegen Qinlian Decoction on Histopathological Changes in Colon Tissues

**FIGURE 9:**
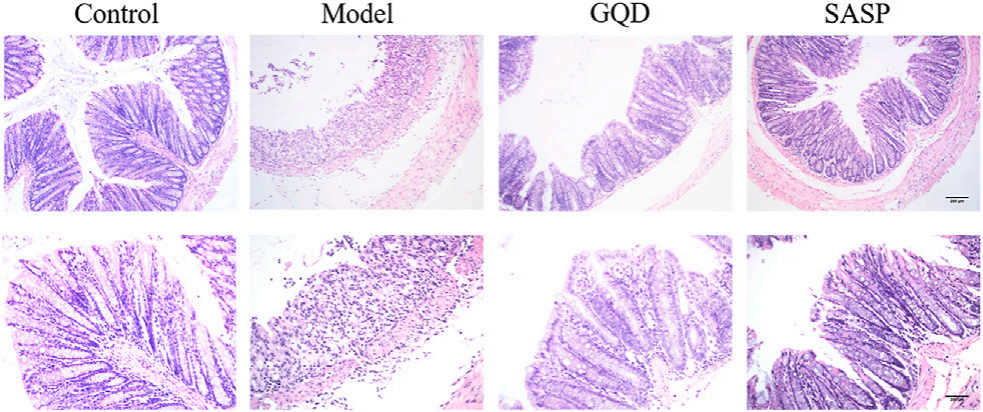
Representative images of colonic tissues with HE staining (× 100 and × 200 magnification).

The diarrhea and stool blood caused by daily DSS administration were accompanied by inflammation in colonic tissues and damage to the intestinal wall. HE staining was applied to observe the pathological changes of intestinal tissue in each group. As shown in Figure 9, the colonic mucosa was intact, and the intestinal epithelial cells and glands were arranged neatly in control group. And severe epithelial cell necrosis, crypt destruction and inflammatory cell infiltration were observed in DSS-treated mice. However, both GQD and SASP treatment could attenuate the histopathological manifestation of colitis. Mild epithelial cells degeneration or necrosis, and a small number of inflammatory cells could be observed in GQD and SASP group.

### Gegen Qinlian Decoction-Treatment Inhibited the Pro-Inflammatory Cytokines in Colon Tissues

**FIGURE 10:**
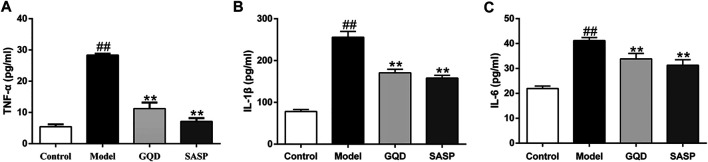
The influence of GQD and SASP on proinflammatory cytokines TNFα **(A)**, IL-1β **(B)**, and IL-6 **(C)** in the colonic tissues. Values presented as mean ± SEM. ^##^ *p* < 0.01 vs. control; ^**^ *p* < 0.01 vs. model.

As pro-inflammatory factors play important roles in the pathogenesis and progress of UC, we investigated whether GQD possess the anti-inflammatory effect in UC. ELISA essay was performed in colonic samples of all the experimental groups. As shown in Figure 10, DSS significantly evoked the expression of pro-inflammatory cytokines including TNF-α, IL-1β, and IL-6 compared with those in sham group (*p* < 0.01). However, the administration of GQD and SASP inhibited the content of TNF-α, IL-1β, and IL-6 significantly than that of the model group (*p* < 0.01). These data demonstrated that GQD and SASP could inhibit the inflammatory reaction induced by DSS in UC.

### Gegen Qinlian Decoction Ameliorated Dextran Sulfate Sodium-Induced Inflammation Through EGFR/PI3K/AKT Signaling Pathway

**FIGURE 11:**
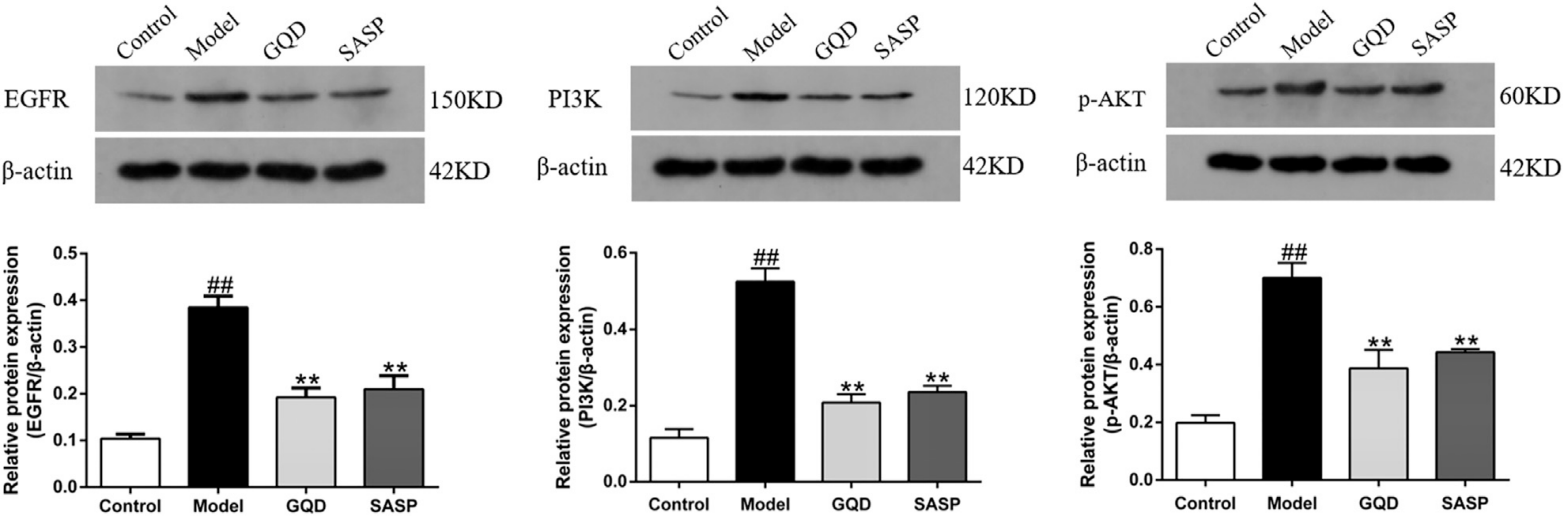
Western blot and quantitative analysis of EGFR, PI3K and *p*-AKT in the colon tissues. Values presented as mean ± SEM. ^##^ *p* < 0.01 vs. control; ^**^ *p* < 0.01 vs. model.

Based on numerous studies and the network pharmacology analysis, inflammation plays an essential role in the pathogenesis and process of UC, and the inflammatory response may partially mediate by EGFR/PI3K/AKT signaling pathway (Neurath, 2014). Thereby, we investigated whether GQD could exert therapeutic effects on UC by inhibiting inflammation via regulating the expression of EGFR, PI3K, and *p*-AKT. As shown in Figure 11, western blotting results showed that DSS induced a remarkably increase in the protein expression of EGFR, PI3K, and *p*-AKT in colon tissue, which indicates the activation of EGFR/PI3K/AKT signaling pathway in UC. Notably, the elevation of these protein targets was blocked by GQD treatment. Similarly, administration of SASP downregulated the protein expression of EGFR, PI3K, and *p*-AKT as well. These results demonstrated that GQD protects against UC partially through inhibiting the activation of EGFR/PI3K/AKT signaling pathway.

## Discussion

UC is a common intestinal inflammatory disease. Presently, numerous studies have demonstrated that GQD, as a classical complementary and alternative TCM prescription, exerted acclaimed therapeutic effects against UC (Xu et al., 2015; Li et al., 2016). Whereas, these clinical or experimental trials were designed on the basis of traditional research model of “one drug, one target”, ignoring the complexity of biological systems. It is well known that botanical medicines usually contain a large group of chemical components, which may act synergistically to achieve the therapeutic effects (Qiao et al., 2016). Thus, to uncover the underlying mechanisms of the therapeutic effects of GQD on UC in a systemic perspective, the present study employed network pharmacological strategy. By means of active compounds screening, drug targeting, and pathway enrichment analysis, network pharmacological analysis of GQD identified four botanical drugs, 77 compounds, and 59 hub targets, which were significantly enriched in several pathways related to UC such as HIF-1 signaling pathway, PI3K-AKT signaling pathway and TNF signaling pathway. In addition, molecular docking results demonstrated that the crucial active compounds in GQD exhibited good binding affinity to the hub target. The putative active components and multi-targets mechanisms of GQD in the treatment of UC were comprehensively elucidated in the present study, which provided theoretic evidence for the clinical application of GQD on UC treatment.

Among the identified compounds linked to the network, most chemical ingredients in GQD related to UC could be classified into flavonoids, alkaloids, and triterpenoid saponins. Baicalein and wogonin (a flavonoid), the major bioactive compounds isolated from HQ, had a better absorption effect. It has been reported that baicalein possesses potent anti-inflammatory and anti-colitis effect via suppressing the activity of TNF-α and interleukin IL-1β (He et al., 2015; Luo et al., 2017), as well as inhibiting the expression of NF-κB and STAT3 signaling pathways (Dong et al., 2015), while the wogonin exerts a dramatically preventive effect on colitis-associated cancer with its anti-angiogenesis activity through inhibiting secretion of VEGF and accelerated the degradation of HIF-1α (Song et al., 2013; Sun et al., 2015). Additionally, formononetin (an isoflavone) was also recognized as one of potential ingredients in the treatment of UC, which might possess the prominent anti-inflammation and antiproliferative effect on human colorectal cancer by downregulating the expression of HIF-1α and inflammatory cytokines (Huang et al., 2015; Ong et al., 2019), such as TNF-α, NF-κB (Wang et al., 2012). Glabridin, another isoflavonoid retrieved from GC, associated with a wide range of biological properties such as antioxidant, anti-inflammatory and antibacterial activities (Simmler et al., 2013). In addition, berberine possessed relatively high bioactivity and showed the high degree number of interactions linked to hub targets. It was a quaternary ammonium alkaloid from HL that exerted beneficial effects against UC via suppressing the IL-6/STAT3/NF-κB signaling pathway *in vivo* (Zhang et al., 2017; Zhu et al., 2019). According to previous research, the anti-inflammatory effect of berberine was mediated through COX-2 regulation (Kuo et al., 2004). Epiberberine is also a protoberberine alkaloid with antibacterial activities and synergistic effects for berberine (Luo et al., 2013; Tan et al., 2017). Moreover, puerarin, one of the main isoflavonoid components in *Pueraria lobata (Willd.) Ohwi*, has been known to possess anti-inflammatory and antioxidant effects in UC via down-regulating the expression of nuclear factor-κB and the secretion of pro-inflammatory mediators (Jeon et al., 2020). Overall, the above findings indicate that multi-compounds of GQD may conjointly execute the beneficial effects for UC. And the HPLC results in the present study also showed the existence and contents of puerarin, baicalein, berberine, and glabridin in GQD, which provided the pharmacological evidence for GQD to be used as an agent in the UC treatment. Besides the therapeutic effects of important compounds mentioned above, multiple actions and pharmacological efficacy of comprehensive ingredients in GQD have not been well evaluated yet, which is a challenge in pharmacology study due to the complex composition of compounds, synergetic effects and multi-targets of compounds in botanical drugs (Heinrich et al., 2020). And in the present study, numerous targets and pathways were identified to be relate to multiple compounds from different botanical drugs in GQD, which revealed the synergistic property of the compounds in GQD for the treatment of UC.

Among the main putative target mapped to compounds in the C-T network, many targets (ESR1, EGFR, SRC, MMP2, GSK3B, and PTGS2) with higher degree to compound might play a crucial role in the process of UC treatment. ESR1, an estrogen receptor, has been identified as a potential marker for identifying individuals at increased risk of neoplasia among those with long-standing and extensive UC (Principi et al., 2014). EGFR, the epidermal growth factor receptor, is one of the major proteins regulating cell proliferation and survival on gut mucosal (Yarden and Pines, 2012). The binding of EGF at the enterocyte surface induces the dimerization of EGFR and tyrosine autophosphorylation, of which the formation initiates several intracellular signaling pathways including PI3K/AKT signaling pathway, and Ras/ERK signaling pathway (Wang, 2017). The GSK3B, as the components of the PI3K pathway, also can be initiated by chronic inflammation and the increased turnover of epithelial cells in the development of UC (Rogler, 2014; Setia et al., 2014). And the expression level of main target MMP2 also has been found to be closely related with TNF-α in colonic mucosa subjected to UC inflammation (Mao et al., 2012). In this study, we speculated that above targets closely related to GQD may be important functional targets for the treatment of UC. Besides, according to GO enrichment and KEGG pathway enrichment, the network analysis revealed that the GQD may exert a therapeutic effect to UC via many cellular processes, such as the regulation of inflammatory response, cell proliferation, angiogenesis and apoptosis. Moreover, among the enriched pathways linked to the hub targets, multiple pathways, such as PI3K-AKT signaling pathway, HIF-1 signaling pathway, VEGF signaling pathway, TNF signaling pathway, and Ras signaling pathway may be closely related to the pathogenesis of UC.

Dysregulated inflammatory response has been demonstrated to play a crucial role in the initiation, development, and progression of UC (Neurath, 2014). Inflammation and inflammatory cytokine in intestinal epithelial cells, and inflammatory cells were recognized as a risk factor for chronic inflammation to tumorigenesis (Yao et al., 2019). Moreover, colorectal cancer is a well-recognized complication of UC. And patients with long-standing chronic inflammation have an increased risk of potential carcinogenesis (Rubin et al., 2012). In UC, the disturbed intestinal epithelial cell generated the mucosal barrier dysfunction and the development of ulcers, leading to cancer occurrence (Yang et al., 2009). In the network pharmacology study, we found that many active components, prediction targets and pathways related to GQD were involved in the inflammation, angiogenesis, cell proliferation and apoptosis, which are closely related to cell carcinogenesis in UC. Specifically, the role of EGFR in inflammatory pathologies of colon is well known, and the expression of EGFR has been found upregulate in experimental colitis and patients with UC, then regulates cytokines production and intestinal inflammation (Lu et al., 2014). Moreover, PI3K is one of the EGFR targets, and the abnormality of PI3K/AKT signaling pathway is considered to be related to the onset of UC, as well as UC-induced colon cancer (Jiang et al., 2019). PI3K-AKT pathway is tightly related to the regulation of inflammatory response in the progression of UC. The abnormal activation of PI3K/AKT signaling pathway in UC has been demonstrated to enhance the expression and secretion of proinflammatory cytokines, such as TNF-α, IL-1β, and IL-6 (Wu et al., 2013; Jiang et al., 2019). However, TNF-a is a pro-inflammatory cytokine central to the pathogenesis of UC, which has a direct effect on the expression and organization of tight junction proteins, resulting in loss of tight junction functions, increased epithelial permeability and the induction of an inflammatory response (Leppkes et al., 2014). Hence, anti-TNF therapies is widely used in clinic as an appropriate way in the management of UC (Lawrance, 2015). Especially, TNF signaling pathway exerts a broad spectrum of activities, including regulation of inflammation and the migration of intestinal epithelial cells (Armaka et al., 2008; Schliemann et al., 2011), and it has been confirmed that ErbB signaling can be activated by the phosphorylation of TNF-α-induced receptor, which modulates the intestinal wound healing of inflammatory bowel disease (Frey et al., 2009). And PI3K/AKT signaling pathway is active in cells infiltrating inflamed human colon tissue via the participation of PTEN, GSK3B and mTOR, which may lead to over-transcription of downstream target genes, proliferation of intestinal epithelial cells, and even dysplasia and cancerization (Castellano and Downward, 2011; Matsuda et al., 2013; Chen et al., 2015). In addition, HIF-1, a transcription factor that regarded as the master regulator of oxygen homeostasis, is involved in the expression of a broad genetic program, including toll-like receptors (TLR)-mediated inflammatory pathway, which can trigger the activation of transcription factors (such as NF-κB) and induce TNF-a secretion (Jung et al., 2003; Kim et al., 2012). It has been reported that HIF-1 play an important role in vascular remodeling at sites of intestinal injury and inflammation of IBD, by regulating angiogenic genes such as VEGF (Feinman et al., 2010; Bakirtzi et al., 2014), a major paracrine growth factor involved in blood vessel development, which has been unregulated in active UC and UC-associated cancer (Tolstanova et al., 2009). Meanwhile, the fundamental regulator of angiogenesis and vascular permeability of VEGF has been found, which indicates that VEGF may also has proinflammatory properties (Chidlow et al., 2006). VEGF signaling pathway can be promoted by increased many inflammatory cells infiltrating ulcerative mucosa. And the increased leukocyte infiltration would contribute to inflammation initiation and subsequent tissue damage which is a characteristic feature of UC (Goebel et al., 2006). Therefore, we hypothesize that GQD may exert its therapeutic effects on UC by decreasing the inflammation response via regulating EGFR/PI3K/AKT signaling pathway.

To examine the effect of GQD on the inflammatory response in UC, as well as the role of EGFR-regulated PI3K/AKT signaling pathway in DSS-induced UC, we evaluated colon inflammation and injury in mice administrated with DSS. DSS-treated mice in model group exhibited more severe injury and acute colitis, while these abnormalities were ameliorated by GQD or SASP administration. We found that GQD and SASP could alleviate the shortening of the colon length, the DAI parameters, the pathological changes and the secretion of inflammatory cytokines. Moreover, the results indicated that EGFR/PI3K/AKT signaling pathway was activated abnormally in model group and played an important role in inflammation, consequently, a series of inflammatory responses and mucosal damage occurs, resulting in the development of UC. However, the expression of EGFR, PI3K and *p*-AKT in the colon tissues was significantly decreased in the GQD and SASP groups compared to model group, suggesting the protective effects of GQD and SASP in the treatment of UC. From the integrated drug target prediction, pathway enrichment analysis and *in vivo* experiment, the results demonstrated that the effective downregulation of the expression of EGFR, PI3K, and *p*-AKT by the administration of GQD and SASP may contribute to ameliorating UC.

The limitation of this study is lacking standardization of active compounds in GQD. However, it has been demonstrated that the governing authorities’ rules on market entry of TCM products is primarily focused on the quality, safety and efficacy of TCM (Li et al., 2018). Compared with chemical drugs and biological products, the quality control of TCM is much more complicated and difficult because of the extremely large amounts of bioactive components and diverse synergistic effects. Standardization of TCM is an essential part of the modernization of TCM, which has been a topic of discussion over the past few decades in China with the goal to promote advances in TCM (Song et al., 2011). With regards to quality, international consensus is that all the TCM products must meet certain qualitative and quantitative quality standards that demonstrated authentication, identification and chemical composition (Qu et al., 2019), and several national standards of TCM has been proposed in China over the past few decades, *Chinese Pharmacopoeia* is the cornerstone of national standards for TCM (Song et al., 2011). The considerations for quality control should mainly include the following aspects: the source control of natural raw material, standardization of decoction pieces processing, in-process control of manufacturing, and specification that fit the characteristics of TCM. In addition, for the selection of quantitative control indicators, the active ingredients that relate to clinical function indications is considered, and when the active ingredients are unclear, representative components should be chosen (Qu et al., 2019). Development of the qualitative and quantitative analytic method to capture the fingerprint and content information of multiple chemical markers is the basis for standardization of TCM. Recently, a concept of “quality marker” (Q-marker) was proposed. Q-marker-base fingerprint and multi-component determination could contribute to the construction of more scientific quality control system of TCM (Yang et al., 2017). TCM standardization is an inevitable trend for the purpose of comprehensive development of TCM, and many measures should be taken to further promote the standardization of TCM.

Most recently, the draft of *Network Pharmacology Evaluation Method Guidance* was proposed, which addressed the data collection, network analysis, experiment verification principles, procedures, and evaluation indicators in network pharmacology research (Li, 2021). According to the guidance, there are multiple problems in the current network pharmacology studies, such as uneven research quality, lack of data standardization, and insufficient scientific verification. Here, we use the evaluation methods from three aspects–reliability, standardization, and rationality to assess the quality of our present study and find that it basically meets the evaluation requirement. Biological functional prediction is an important part in network pharmacology. The inflammatory response, regulation of apoptotic process, protein phosphorylation, and cell proliferation have been identified as important biological functions according to our prediction, while we mainly focused on inflammatory response in this paper, and in the future we will carry out further experiments to probe other possible predicted functions and mechanisms.

## Conclusion

In summary, the combination of network pharmacological analysis in silico and *in vivo* experiment indicated that GQD could effectively ameliorate DSS-induced colitis, and the therapeutic effect of GQD was associated with its synergic modulation on inflammation suppression via EGFR/PI3K/AKT signaling pathway. Therefore, we suggest that GQD could be developed as a promising therapy for UC, and it provided novel indications for further mechanistic studies of GQD on UC treatment. Our study also suggested that the network pharmacology prediction may exert as a useful tool to characterize the pharmacological mechanisms of TCM in detail.

## Data Availability

The raw data supporting the conclusions of this article will be made available by the authors, without undue reservation, to any qualified researcher.

## Abbreviations

UC, ulcerative colitis; GQD, gegen qinlian decoction; TCMSP, traditional chinese medicine systems pharmacology database and analysis platform; BATMAN-TCM, bioinformatics analysis tool for molecular mechANism of TCM; TTD, therapeutic target database; OMIM, online mendelian inheritance in man; PPI, protein-protein interaction; GO, dene ontology; KEGG, kyoto encyclopedia of genes and genomes; SASP, sulfasalazine; IBD, inflammatory bowel disease; TCM, traditional chinese medicine; GG*, pueraria lobata (Willd.) ohwi* (Ge-Gen in Chinese); HQ*, scutellaria baicalensis georgi* (Huang-Qin in Chinese); HL*, coptis chinensis franch* (Huang-Lian in Chinese); GC*, glycyrrhiza uralensis fisch* (Gan-Cao in Chinese); OB, oral bioavailability; Caco-2, Caco-2 cell permeability; DL, drug-likeness; DAVID, database for annotation, visualization, and integrated discovery; DSS, dextran sulfate sodium; HPLC, high-performance liquid chromatography; TNF-α, tumor necrosis factor-α; IL-1β, interleukin-1 beta; IL-6, interleukin-6; EGFR, epidermal growth factor receptor erbB1; PI3K, phosphoinositide 3-kinase; AKT, serine/threonine-protein kinase akt-1; Q-marker, quality marker.
